# Perspective: Dimensions of the scientific method

**DOI:** 10.1371/journal.pcbi.1007279

**Published:** 2019-09-12

**Authors:** Eberhard O. Voit

**Affiliations:** Department of Biomedical Engineering, Georgia Institute of Technology and Emory University, Atlanta, Georgia, United States of America; University of Virginia, UNITED STATES

## Abstract

The scientific method has been guiding biological research for a long time. It not only prescribes the order and types of activities that give a scientific study validity and a stamp of approval but also has substantially shaped how we collectively think about the endeavor of investigating nature. The advent of high-throughput data generation, data mining, and advanced computational modeling has thrown the formerly undisputed, monolithic status of the scientific method into turmoil. On the one hand, the new approaches are clearly successful and expect the same acceptance as the traditional methods, but on the other hand, they replace much of the hypothesis-driven reasoning with inductive argumentation, which philosophers of science consider problematic. Intrigued by the enormous wealth of data and the power of machine learning, some scientists have even argued that significant correlations within datasets could make the entire quest for causation obsolete. Many of these issues have been passionately debated during the past two decades, often with scant agreement. It is proffered here that hypothesis-driven, data-mining–inspired, and “allochthonous” knowledge acquisition, based on mathematical and computational models, are vectors spanning a 3D space of an expanded scientific method. The combination of methods within this space will most certainly shape our thinking about nature, with implications for experimental design, peer review and funding, sharing of result, education, medical diagnostics, and even questions of litigation.

## The traditional scientific method: Hypothesis-driven deduction

Research is the undisputed core activity defining science. Without research, the advancement of scientific knowledge would come to a screeching halt. While it is evident that researchers look for new information or insights, the term “research” is somewhat puzzling. Never mind the prefix “re,” which simply means “coming back and doing it again and again,” the word “search” seems to suggest that the research process is somewhat haphazard, that not much of a strategy is involved in the process. One might argue that research a few hundred years ago had the character of hoping for enough luck to find something new. The alchemists come to mind in their quest to turn mercury or lead into gold, or to discover an elixir for eternal youth, through methods we nowadays consider laughable.

Today’s sciences, in stark contrast, are clearly different. Yes, we still try to find something new—and may need a good dose of luck—but the process is anything but unstructured. In fact, it is prescribed in such rigor that it has been given the widely known moniker “scientific method.” This scientific method has deep roots going back to Aristotle and Herophilus (approximately 300 BC), Avicenna and Alhazen (approximately 1,000 AD), Grosseteste and Robert Bacon (approximately 1,250 AD), and many others, but solidified and crystallized into the gold standard of quality research during the 17th and 18th centuries [1–7]. In particular, Sir Francis Bacon (1561–1626) and René Descartes (1596–1650) are often considered the founders of the scientific method, because they insisted on careful, systematic observations of high quality, rather than metaphysical speculations that were *en vogue* among the scholars of the time [1, 8]. In contrast to their peers, they strove for objectivity and insisted that observations, rather than an investigator’s preconceived ideas or superstitions, should be the basis for formulating a research idea [7, 9].

Bacon and his 19th century follower John Stuart Mill explicitly proposed gaining knowledge through inductive reasoning: Based on carefully recorded observations, or from data obtained in a well-planned experiment, generalized assertions were to be made about similar yet (so far) unobserved phenomena [7]. Expressed differently, inductive reasoning attempts to derive general principles or laws directly from empirical evidence [10]. An example is the 19th century epigram of the physician Rudolf Virchow, *Omnis cellula e cellula*. There is no proof that indeed “every cell derives from a cell,” but like Virchow, we have made the observation time and again and never encountered anything suggesting otherwise.

In contrast to induction, the widely accepted, traditional scientific method is based on formulating and testing hypotheses. From the results of these tests, a deduction is made whether the hypothesis is presumably true or false. This type of hypotheticodeductive reasoning goes back to William Whewell, William Stanley Jevons, and Charles Peirce in the 19th century [1]. By the 20th century, the deductive, hypothesis-based scientific method had become deeply ingrained in the scientific psyche, and it is now taught as early as middle school in order to teach students valid means of discovery [8, 11, 12]. The scientific method has not only guided most research studies but also fundamentally influenced how we think about the process of scientific discovery.

Alas, because biology has almost no general laws, deduction in the strictest sense is difficult. It may therefore be preferable to use the term abduction, which refers to the logical inference toward the most plausible explanation, given a set of observations, although this explanation cannot be proven and is not necessarily true.

**Fig 1:**
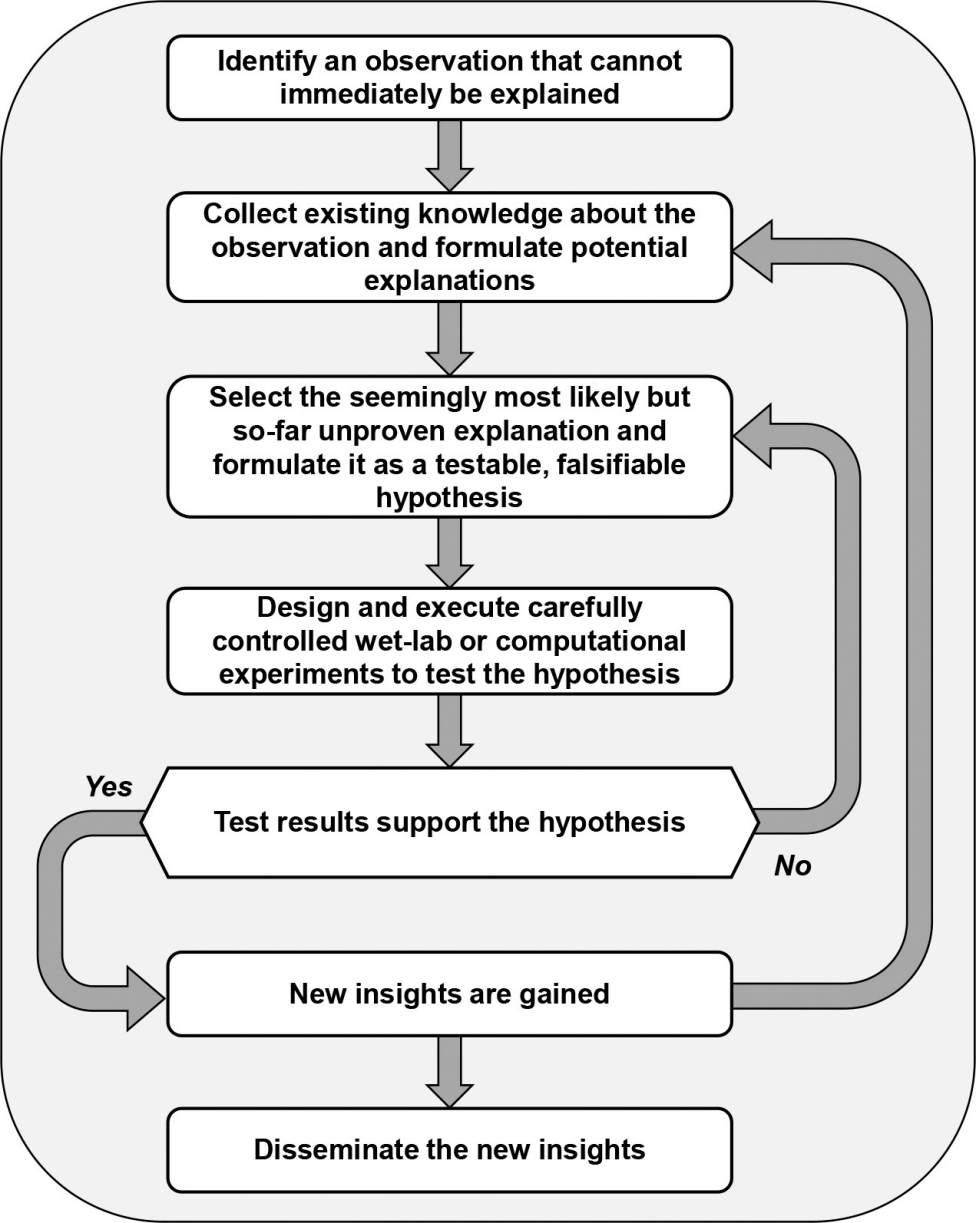
Traditional scientific method: Hypothesis-based deduction. The central concept of the traditional scientific method is a falsifiable hypothesis regarding some phenomenon of interest. This hypothesis is to be tested experimentally or computationally. The test results support or refute the hypothesis, triggering a new round of hypothesis formulation and testing.

Over the decades, the hypothesis-based scientific method did experience variations here and there, but its conceptual scaffold remained essentially unchanged (Fig 1). Its key is a process that begins with the formulation of a hypothesis that is to be rigorously tested, either in the wet lab or computationally; nonadherence to this principle is seen as lacking rigor and can lead to irreproducible results [1, 13–15].

Going further, the prominent philosopher of science Sir Karl Popper argued that a scientific hypothesis can never be verified but that it can be disproved by a single counterexample. He therefore demanded that scientific hypotheses had to be falsifiable, because otherwise, testing would be moot [16, 17] (see also [18]). As Gillies put it, “successful theories are those that survive elimination through falsification” [19]. Kelley and Scott agreed to some degree but warned that complete insistence on falsifiability is too restrictive as it would mark many computational techniques, statistical hypothesis testing, and even Darwin’s theory of evolution as nonscientific [20].

While the hypothesis-based scientific method has been very successful, its exclusive reliance on deductive reasoning is dangerous because according to the so-called Duhem–Quine thesis, hypothesis testing always involves an unknown number of explicit or implicit assumptions, some of which may steer the researcher away from hypotheses that seem implausible, although they are, in fact, true [21]. According to Kuhn, this bias can obstruct the recognition of paradigm shifts [22], which require the rethinking of previously accepted “truths” and the development of radically new ideas [23, 24]. The testing of simultaneous alternative hypotheses [25–27] ameliorates this problem to some degree but not entirely.

The traditional scientific method is often presented in discrete steps, but it should really be seen as a form of critical thinking, subject to review and independent validation [8]. It has proven very influential, not only by prescribing valid experimentation, but also for affecting the way we attempt to understand nature [18], for teaching [8, 12], reporting, publishing, and otherwise sharing information [28], for peer review and the awarding of funds by research-supporting agencies [29, 30], for medical diagnostics [7], and even in litigation [31].

### A second dimension of the scientific method: Data-mining–inspired induction

A major shift in biological experimentation occurred with the–omics revolution of the early 21st century. All of a sudden, it became feasible to perform high-throughput experiments that generated thousands of measurements, typically characterizing the expression or abundances of very many—if not all—genes, proteins, metabolites, or other biological quantities in a sample.

**Fig 2:**
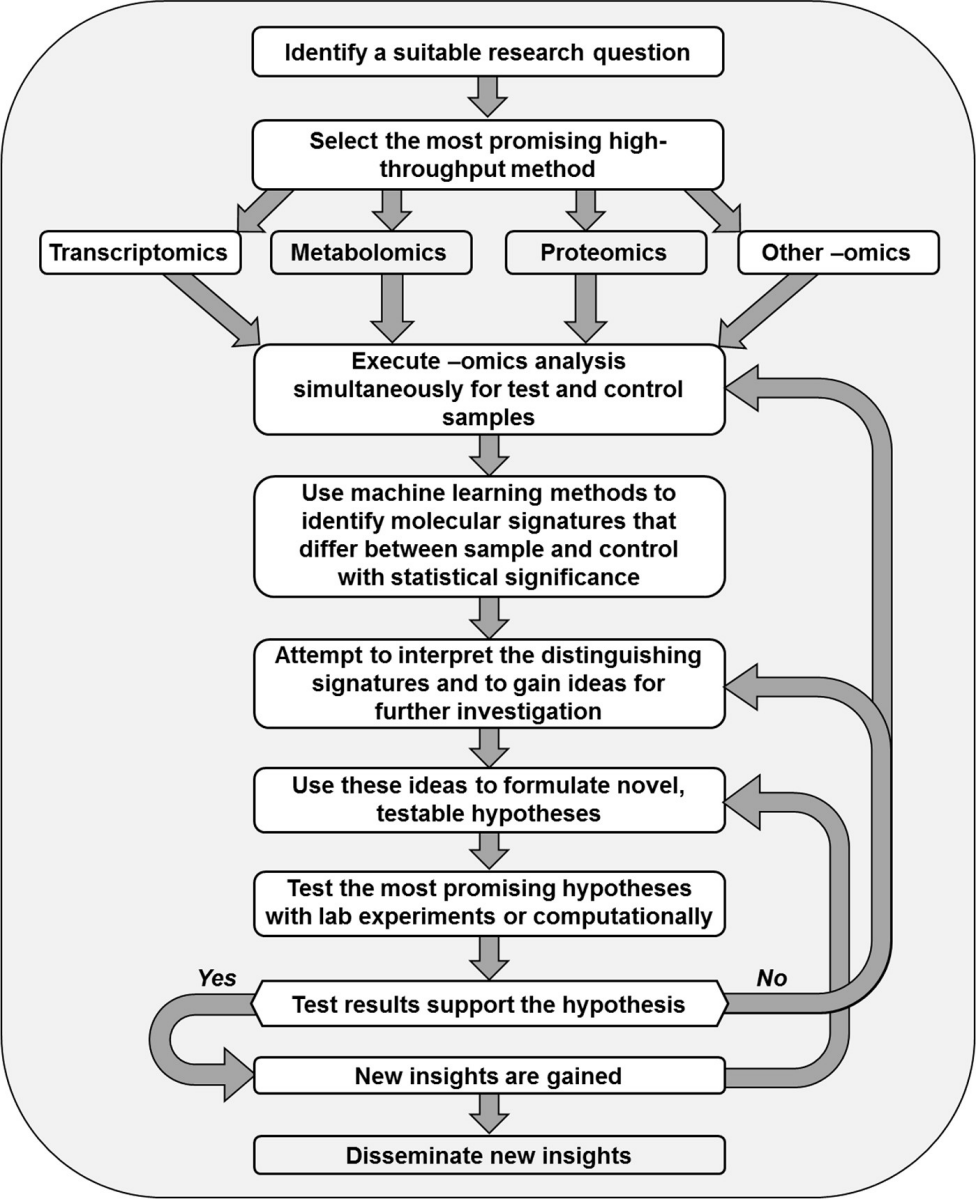
Dimension of data-mining–inspired induction. Data-driven research begins with an untargeted exploration, in which the data speak for themselves. Machine learning extracts patterns from the data, which suggest hypotheses that are to be tested in the lab or computationally.

The strategy of measuring large numbers of items in a nontargeted fashion is fundamentally different from the traditional scientific method and constitutes a new, second dimension of the scientific method. Instead of hypothesizing and testing whether gene X is up-regulated under some altered condition, the leading question becomes which of the thousands of genes in a sample are up- or down-regulated. This shift in focus elevates the data to the supreme role of revealing novel insights by themselves (Fig 2). As an important, generic advantage over the traditional strategy, this second dimension is free of a researcher’s preconceived notions regarding the molecular mechanisms governing the phenomenon of interest, which are otherwise the key to formulating a hypothesis. The prominent biologists Patrick Brown and David Botstein commented that “the patterns of expression will often suffice to begin de novo discovery of potential gene functions” [32].

This data-driven, discovery-generating approach is at once appealing and challenging. On the one hand, very many data are explored simultaneously and essentially without bias. On the other hand, the large datasets supporting this approach create a genuine challenge to understanding and interpreting the experimental results because the thousands of data points, often superimposed with a fair amount of noise, make it difficult to detect meaningful differences between sample and control. This situation can only be addressed with computational methods that first “clean” the data, for instance, through the statistically valid removal of outliers, and then use machine learning to identify statistically significant, distinguishing molecular profiles or signatures. In favorable cases, such signatures point to specific biological pathways, whereas other signatures defy direct explanation but may become the launch pad for follow-up investigations [33].

Today’s scientists are very familiar with this discovery-driven exploration of “what’s out there” and might consider it a quaint quirk of history that this strategy was at first widely chastised and ridiculed as a “fishing expedition” [30, 34]. Strict traditionalists were outraged that rigor was leaving science with the new approach and that sufficient guidelines were unavailable to assure the validity and reproducibility of results [10, 35, 36].

From the view point of philosophy of science, this second dimension of the scientific method uses inductive reasoning and reflects Bacon’s idea that observations can and should dictate the research question to be investigated [1, 7]. Allen [36] forcefully rejected this type of reasoning, stating “the thinking goes, we can now expect computer programs to derive significance, relevance and meaning from chunks of information, be they nucleotide sequences or gene expression profiles… In contrast with this view, many are convinced that no purely logical process can turn observation into understanding.” His conviction goes back to the 18th century philosopher David Hume and again to Popper, who identified as the overriding problem with inductive reasoning that it can never truly reveal causality, even if a phenomenon is observed time and again [16, 17, 37, 38]. No number of observations, even if they always have the same result, can guard against an exception that would violate the generality of a law inferred from these observations [1, 35]. Worse, Popper argued, through inference by induction, we cannot even know the probability of something being true [10, 17, 36].

Others argued that data-driven and hypothesis-driven research actually do not differ all that much in principle, as long as there is cycling between developing new ideas and testing them with care [27]. In fact, Kell and Oliver [34] maintained that the exclusive acceptance of hypothesis-driven programs misrepresents the complexities of biological knowledge generation. Similarly refuting the prominent rule of deduction, Platt [26] and Beard and Kushmerick [27] argued that repeated inductive reasoning, called strong inference, corresponds to a logically sound decision tree of disproving or refining hypotheses that can rapidly yield firm conclusions; nonetheless, Platt had to admit that inductive inference is not as certain as deduction, because it projects into the unknown. Lander compared the task of obtaining causality by induction to the problem of inferring the design of a microprocessor from input-output readings, which in a strict sense is impossible, because the microprocessor could be arbitrarily complicated; even so, inference often leads to novel insights and therefore is valuable [39].

An interesting special case of almost pure inductive reasoning is epidemiology, where hypothesis-driven reasoning is rare and instead, the fundamental question is whether data-based evidence is sufficient to associate health risks with specific causes [31, 34].

Recent advances in machine learning and “big-data” mining have driven the use of inductive reasoning to unprecedented heights. As an example, machine learning can greatly assist in the discovery of patterns, for instance, in biological sequences [40]. Going a step further, a pithy article by Andersen [41] proffered that we may not need to look for causality or mechanistic explanations anymore if we just have enough correlation: “With enough data, the numbers speak for themselves, correlation replaces causation, and science can advance even without coherent models or unified theories.”

Of course, the proposal to abandon the quest for causality caused pushback on philosophical as well as mathematical grounds. Allen [10, 35] considered the idea “absurd” that data analysis could enhance understanding in the absence of a hypothesis. He felt confident “that even the formidable combination of computing power with ease of access to data cannot produce a qualitative shift in the way that we do science: the making of hypotheses remains an indispensable component in the growth of knowledge” [36]. Succi and Coveney [42] refuted the “most extravagant claims” of big-data proponents very differently, namely by analyzing the theories on which machine learning is founded. They contrasted the assumptions underlying these theories, such as the law of large numbers, with the mathematical reality of complex biological systems. Specifically, they carefully identified genuine features of these systems, such as nonlinearities, nonlocality of effects, fractal aspects, and high dimensionality, and argued that they fundamentally violate some of the statistical assumptions implicitly underlying big-data analysis, like independence of events. They concluded that these discrepancies “may lead to false expectations and, at their nadir, even to dangerous social, economical and political manipulation.” To ameliorate the situation, the field of big-data analysis would need new strong theorems characterizing the validity of its methods and the numbers of data required for obtaining reliable insights. Succi and Coveney go as far as stating that too many data are just as bad as insufficient data [42].

While philosophical doubts regarding inductive methods will always persist, one cannot deny that -omics-based, high-throughput studies, combined with machine learning and big-data analysis, have been very successful [43]. Yes, induction cannot truly reveal general laws, no matter how large the datasets, but they do provide insights that are very different from what science had offered before and may at least suggest novel patterns, trends, or principles. As a case in point, if many transcriptomic studies indicate that a particular gene set is involved in certain classes of phenomena, there is probably some truth to the observation, even though it is not mathematically provable. Kepler’s laws of astronomy were arguably derived solely from inductive reasoning [34].

Notwithstanding the opposing views on inductive methods, successful strategies shape how we think about science. Thus, to take advantage of all experimental options while ensuring quality of research, we must not allow that “anything goes” but instead identify and characterize standard operating procedures and controls that render this emerging scientific method valid and reproducible. A laudable step in this direction was the wide acceptance of “minimum information about a microarray experiment” (MIAME) standards for microarray experiments [44].

### A third dimension of the scientific method: Allochthonous reasoning

Parallel to the blossoming of molecular biology and the rapid rise in the power and availability of computing in the late 20th century, the use of mathematical and computational models became increasingly recognized as relevant and beneficial for understanding biological phenomena. Indeed, mathematical models eventually achieved cornerstone status in the new field of computational systems biology.

Mathematical modeling has been used as a tool of biological analysis for a long time [27, 45–48]. Interesting for the discussion here is that the use of mathematical and computational modeling in biology follows a scientific approach that is distinctly different from the traditional and the data-driven methods, because it is distributed over two entirely separate domains of knowledge. One consists of the biological reality of DNA, elephants, and roses, whereas the other is the world of mathematics, which is governed by numbers, symbols, theorems, and abstract work protocols. Because the ways of thinking—and even the languages—are different in these two realms, I suggest calling this type of knowledge acquisition “allochthonous” (literally Greek: in or from a “piece of land different from where one is at home”; one could perhaps translate it into modern lingo as “outside one’s comfort zone”). De facto, most allochthonous reasoning in biology presently refers to mathematics and computing, but one might also consider, for instance, the application of methods from linguistics in the analysis of DNA sequences or proteins [49].

One could argue that biologists have employed “models” for a long time, for instance, in the form of “model organisms,” cell lines, or in vitro experiments, which more or less faithfully reflect features of the organisms of true interest but are easier to manipulate. However, this type of biological model use is rather different from allochthonous reasoning, as it does not leave the realm of biology and uses the same language and often similar methodologies.

A brief discussion of three experiences from our lab may illustrate the benefits of allochthonous reasoning. (1) In a case study of renal cell carcinoma, a dynamic model was able to explain an observed yet nonintuitive metabolic profile in terms of the enzymatic reaction steps that had been altered during the disease [50]. (2) A transcriptome analysis had identified several genes as displaying significantly different expression patterns during malaria infection in comparison to the state of health. Considered by themselves and focusing solely on genes coding for specific enzymes of purine metabolism, the findings showed patterns that did not make sense. However, integrating the changes in a dynamic model revealed that purine metabolism globally shifted, in response to malaria, from guanine compounds to adenine, inosine, and hypoxanthine [51]. (3) Data capturing the dynamics of malaria parasites suggested growth rates that were biologically impossible. Speculation regarding possible explanations led to the hypothesis that many parasite-harboring red blood cells might “hide” from circulation and therewith from detection in the blood stream. While experimental testing of the feasibility of the hypothesis would have been expensive, a dynamic model confirmed that such a concealment mechanism could indeed quantitatively explain the apparently very high growth rates [52]. In all three cases, the insights gained inductively from computational modeling would have been difficult to obtain purely with experimental laboratory methods. Purely deductive allochthonous reasoning is the ultimate goal of the search for design and operating principles [53–55], which strives to explain why certain structures or functions are employed by nature time and again. An example is a linear metabolic pathway, in which feedback inhibition is essentially always exerted on the first step [56, 57]. This generality allows the deduction that a so far unstudied linear pathway is most likely (or even certain to be) inhibited at the first step. Not strictly deductive—but rather abductive—was a study in our lab in which we analyzed time series data with a mathematical model that allowed us to infer the most likely regulatory structure of a metabolic pathway [58, 59].

**Fig 3:**
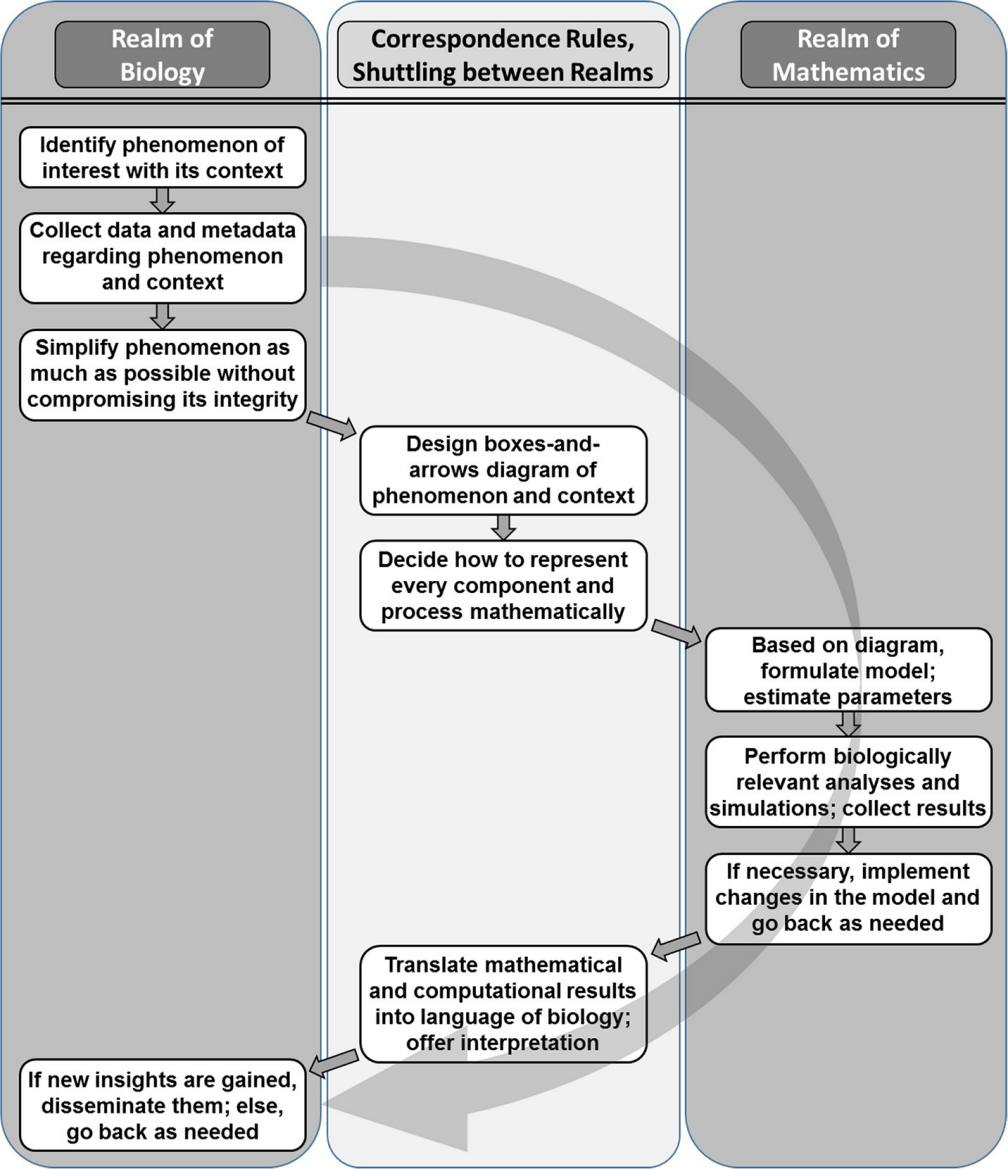
Dimension of allochthonous, model-based reasoning. This mathematical and computational approach is distributed over two realms, which are connected by correspondence rules.

A typical allochthonous investigation begins in the realm of biology with the formulation of a hypothesis (Fig 3). Instead of testing this hypothesis with laboratory experiments, the system encompassing the hypothesis is moved into the realm of mathematics. This move requires two sets of ingredients. One set consists of the simplification and abstraction of the biological system: Any distracting details that seem unrelated to the hypothesis and its context are omitted or represented collectively with other details. This simplification step carries the greatest risk of the entire modeling approach, as omission of seemingly negligible but, in truth, important details can easily lead to wrong results. The second set of ingredients consists of correspondence rules that translate every biological component or process into the language of mathematics [60, 61].

Once the system is translated, it has become an entirely mathematical construct that can be analyzed purely with mathematical and computational means. The results of this analysis are also strictly mathematical. They typically consist of values of variables, magnitudes of processes, sensitivity patterns, signs of eigenvalues, or qualitative features like the onset of oscillations or the potential for limit cycles. Correspondence rules are used again to move these results back into the realm of biology. As an example, the mathematical result that “two eigenvalues have positive real parts” does not make much sense to many biologists, whereas the interpretation that “the system is not stable at the steady state in question” is readily explained. New biological insights may lead to new hypotheses, which are tested either by experiments or by returning once more to the realm of mathematics. The model design, diagnosis, refinements, and validation consist of several phases, which have been discussed widely in the biomathematical literature. Importantly, each iteration of a typical modeling analysis consists of a move from the biological to the mathematical realm and back.

The reasoning within the realm of mathematics is often deductive, in the form of an Aristotelian syllogism, such as the well-known “All men are mortal; Socrates is a man; therefore, Socrates is mortal.” However, the reasoning may also be inductive, as it is the case with large-scale Monte-Carlo simulations that generate arbitrarily many “observations,” although they cannot reveal universal principles or theorems. An example is a simulation randomly drawing numbers in an attempt to show that every real number has an inverse. The simulation will always attest to this hypothesis but fail to discover the truth because it will never randomly draw 0. Generically, computational models may be considered sets of hypotheses, formulated as equations or as algorithms that reflect our perception of a complex system [27].

### Impact of the multidimensional scientific method on learning

Almost all we know in biology has come from observation, experimentation, and interpretation. The traditional scientific method not only offered clear guidance for this knowledge gathering, but it also fundamentally shaped the way we think about the exploration of nature. When presented with a new research question, scientists were trained to think immediately in terms of hypotheses and alternatives, pondering the best feasible ways of testing them, and designing in their minds strong controls that would limit the effects of known or unknown confounders. Shaped by the rigidity of this ever-repeating process, our thinking became trained to move forward one well-planned step at a time. This modus operandi was rigid and exact. It also minimized the erroneous pursuit of long speculative lines of thought, because every step required testing before a new hypothesis was formed. While effective, the process was also very slow and driven by ingenuity—as well as bias—on the scientist’s part. This bias was sometimes a hindrance to necessary paradigm shifts [22].

High-throughput data generation, big-data analysis, and mathematical-computational modeling changed all that within a few decades. In particular, the acceptance of inductive principles and of the allochthonous use of nonbiological strategies to answer biological questions created an unprecedented mix of successes and chaos. To the horror of traditionalists, the importance of hypotheses became minimized, and the suggestion spread that the data would speak for themselves [36]. Importantly, within this fog of “anything goes,” the fundamental question arose how to determine whether an experiment was valid.

**Fig 4:**
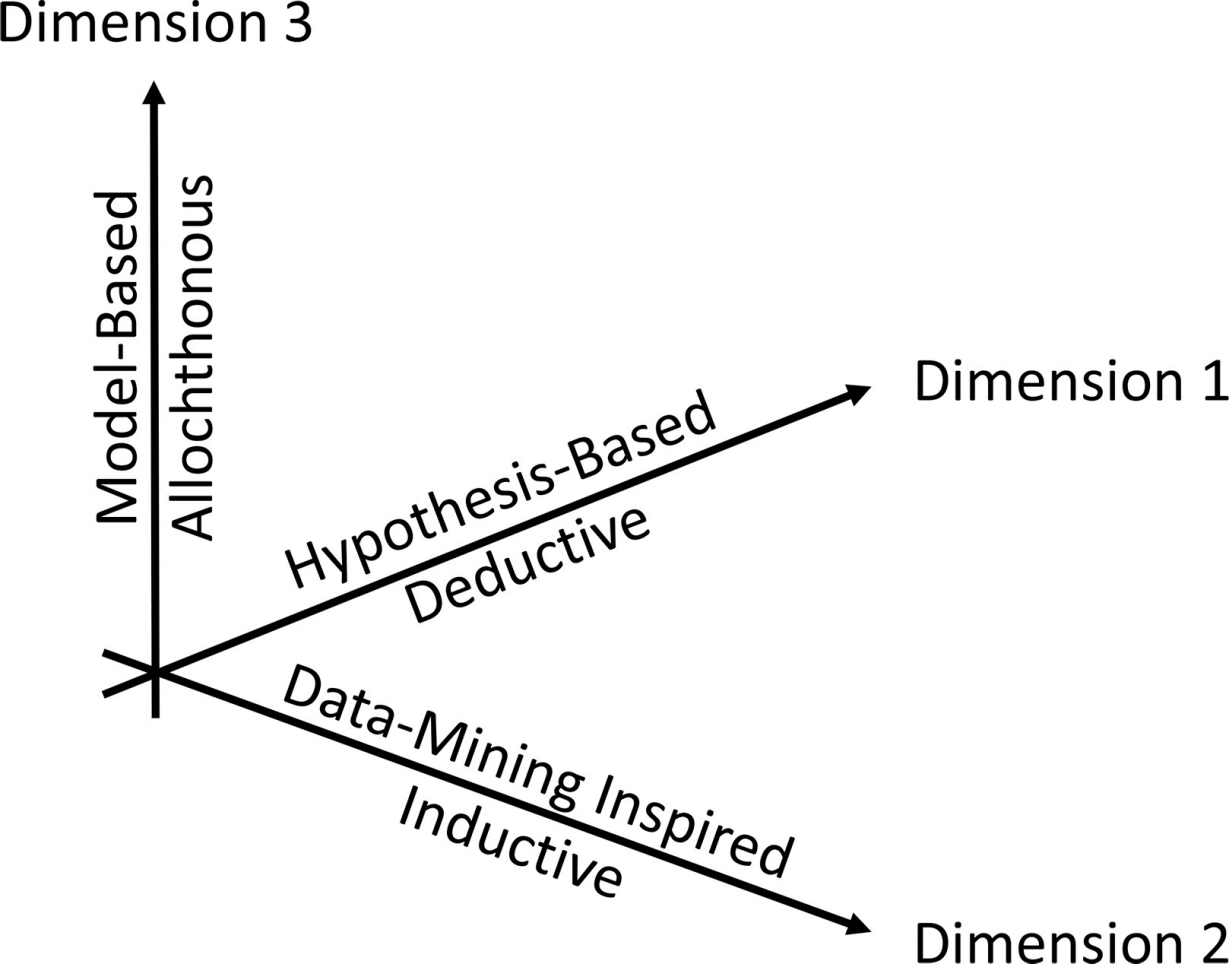
Space of scientific methods. The traditional hypothesis-based deductive scientific method is expanded into a 3D space that allows for synergistic blends of methods that include data-mining–inspired, inductive knowledge acquisition, and mathematical model-based, allochthonous reasoning.

Because agreed-upon operating procedures affect research progress and interpretation, thinking, teaching, and sharing of results, this question requires a deconvolution of scientific strategies. Here I proffer that the single scientific method of the past should be expanded toward a vector space of scientific methods, with spanning vectors that correspond to different dimensions of the scientific method (Fig 4).

Obviously, all three dimensions have their advantages and drawbacks. The traditional, hypothesis-driven deductive method is philosophically “clean,” except that it is confounded by preconceptions and assumptions. The data-mining–inspired inductive method cannot offer universal truths but helps us explore very large spaces of factors that contribute to a phenomenon. Allochthonous, model-based reasoning can be performed mentally, with paper and pencil, through rigorous analysis, or with a host of computational methods that are precise and disprovable [27]. At the same time, they are incomparable faster, cheaper, and much more comprehensive than experiments in molecular biology. This reduction in cost and time, and the increase in coverage, may eventually have far-reaching consequences, as we can already fathom from much of modern physics.

Due to its long history, the traditional dimension of the scientific method is supported by clear and very strong standard operating procedures. Similarly, strong procedures need to be developed for the other two dimensions. The MIAME rules for microarray analysis provide an excellent example [44]. On the mathematical modeling front, no such rules are generally accepted yet, but trends toward them seem to emerge at the horizon. For instance, it seems to be becoming common practice to include sensitivity analyses in typical modeling studies and to assess the identifiability or sloppiness of ensembles of parameter combinations that fit a given dataset well [62, 63].

From a philosophical point of view, it seems unlikely that objections against inductive reasoning will disappear. However, instead of pitting hypothesis-based deductive reasoning against inductivism, it seems more beneficial to determine how the different methods can be synergistically blended (*cf*. [18, 27, 34, 42]) as linear combinations of the three vectors of knowledge acquisition (Fig 4). It is at this point unclear to what degree the identified three dimensions are truly independent of each other, whether additional dimensions should be added [24], or whether the different versions could be amalgamated into a single scientific method [18], especially if it is loosely defined as a form of critical thinking [8]. Nobel Laureate Percy Bridgman even concluded that “science is what scientists do, and there are as many scientific methods as there are individual scientists” [8, 64].

Combinations of the three spanning vectors of the scientific method have been emerging for some time. Many biologists already use inductive high-throughput methods to develop specific hypotheses that are subsequently tested with deductive or further inductive methods [34, 65]. In terms of including mathematical modeling, physics and geology have been leading the way for a long time, often by beginning an investigation in theory, before any actual experiment is performed. It will benefit biology to look into this strategy and to develop best practices of allochthonous reasoning.

The blending of methods may take quite different shapes. Early on, Ideker and colleagues [65] proposed an integrated experimental approach for pathway analysis that offered a glimpse of new experimental strategies within the space of scientific methods. In a similar vein, Covert and colleagues [66] included computational methods into such an integrated approach. Additional examples of blended analyses in systems biology can be seen in other works, such as [43, 67–73]. Generically, it is often beneficial to start with big data, determine patterns in associations and correlations, then switch to the mathematical realm in order to filter out spurious correlations in a high-throughput fashion. If this procedure is executed in an iterative manner, the “surviving” associations have an increased level of confidence and are good candidates for further experimental or computational testing (personal communication from S. Chandrasekaran).

If each component of a blended scientific method follows strict, commonly agreed guidelines, “linear combinations” within the 3D space can also be checked objectively, per deconvolution. In addition, guidelines for synergistic blends of component procedures should be developed. If we carefully monitor such blends, time will presumably indicate which method is best for which task and how the different approaches optimally inform each other. For instance, it will be interesting to study whether there is an optimal sequence of experiments along the three axes for a particular class of tasks. Big-data analysis together with inductive reasoning might be optimal for creating initial hypotheses and possibly refuting wrong speculations (“we had thought this gene would be involved, but apparently it isn’t”). If the logic of an emerging hypotheses can be tested with mathematical and computational tools, it will almost certainly be faster and cheaper than an immediate launch into wet-lab experimentation. It is also likely that mathematical reasoning will be able to refute some apparently feasible hypothesis and suggest amendments. Ultimately, the “surviving” hypotheses must still be tested for validity through conventional experiments. Deconvolving current practices and optimizing the combination of methods within the 3D or higher-dimensional space of scientific methods will likely result in better planning of experiments and in synergistic blends of approaches that have the potential capacity of addressing some of the grand challenges in biology.
